# Chronic exposure to low doses of estradiol-17ß increases blood pressure in young female rats: A possible role for central Endothelin-1

**DOI:** 10.1038/s41598-017-00213-9

**Published:** 2017-03-10

**Authors:** Madhan Subramanian, Sheba M. J. MohanKumar, Priya Balasubramanian, Carrie A. Northcott, Hannah Garver, Gregory D. Fink, P. S. MohanKumar

**Affiliations:** 1 Department of Pathobiology & Diagnostic Investigation, Michigan State University, East Lansing, MI 48824 USA; 2 Department of Veterinary Biosciences and Diagnostic Imaging, University of Georgia, Athens, GA 30602 USA; 3 Department of Pharmacology & Toxicology, Michigan State University, East Lansing, MI 48824 USA

## Abstract

Previously, we demonstrated that chronic exposure to low levels of estradiol-17β (E2) increases mean arterial pressure (MAP) in young female Sprague-Dawley (SD) rats, however, the underlying mechanisms are unclear. Since endothelin-1 (ET-1) is implicated in blood pressure (BP) regulation, we hypothesized that E2’s effects on MAP are mediated through central ET-1. To test this, young female SD rats were either sham implanted or implanted s.c. with slow-release E2 pellets (20 ng/day for 90 days). BP was monitored by telemetry. After 75 days of E2 exposure, ET_A_ antagonist or vehicle was administered i.c.v. After 90 days of E2 exposure, rats were sacrificed, and the paraventricular nucleus (PVN) and rostral ventrolateral medulla (RVLM) were microdissected for gene expression and protein analysis of ET-1 and its receptors. E2 exposure increased MAP after pellet implantation. Gene expression of ET-1 and ET_A_ but not ET_B_ receptors were upregulated in the PVN and RVLM of E2 treated animals. Further, the protein levels of ET_A_ receptor were also increased in the PVN of E2 treated animals. However, i.c.v. infusion of the ET_A_ antagonist did not completely block the increase in blood pressure. Our results suggest that increases in central ET-1 activity could possibly play a role in chronic E2-induced increase in BP but further studies are needed to completely understand the contribution of ET-1 in this phenomenon.

## Introduction

Women on oral contraceptives are known to be at higher risk for developing hypertension compared to non-users^1^. Studies have reported that small increases in blood pressure are apparent in women who are on monophasic pills that contain 30 µg of estrogen for prolonged periods of time^2–4^. Although the magnitude of blood pressure increase is small, large clinical trials have shown that this is associated with a higher rate of progression of coronary atherosclerosis^5^ and development of cardiovascular events^6^. Therefore it is important to understand the mechanisms underlying chronic estrogen-induced increases in blood pressure. Previously, we demonstrated that chronic exposure to low levels of estradiol-17β (E2) increases mean arterial pressure (MAP) in young female rats^7^. We also found that this effect was accompanied by an increase in superoxide levels in the rostral ventrolateral medulla (RVLM). More importantly, treatment with resveratrol, an antioxidant, decreased superoxide levels in the RVLM and reversed E2-induced increase in arterial pressure^7^. Several studies have provided evidence that central endothelin-1 (ET-1) plays a role in the development of neurogenic hypertension directly or indirectly through oxidative stress-related mechanisms^8–12^. The objective of the present study was to identify the role of central ET-1 in chronic E2-induced increase in arterial pressure.

Endothelin-1 (ET-1) is a vasoconstrictor peptide and is known to contribute to the pathogenesis of hypertension in several models of hypertension including deoxycorticosterone acetate (DOCA)-salt^13^ and salt-sensitive hypertension^14^. ET-1 was originally identified in the endothelial cells of the vasculature^15^. Later, ET-1, its receptors ET_A_ and ET_B_, and endothelin converting enzyme (ECE) were identified in brain regions that are involved in cardiovascular regulation such as the paraventricular nucleus (PVN) and the RVLM^16, 17^. Central administration of ET-1 (both i.c.v. and directly into the RVLM) increased MAP and sympathetic nerve activity in several models of hypertension including spontaneously hypertensive rats (SHR), spontaneously hypertensive stroke prone rats (SHR-SP) and DOCA-salt hypertensive rats^18–20^. Microinjection of ET-1 into the PVN also increased renal sympathetic nerve activity (RSNA) and mean arterial pressure (MAP)^21^. Moreover, blockade of ET_A_ receptors reversed ET-1-induced increases in blood pressure^18–20^. Therefore, there is a strong likelihood for ET-1 to play a role in E2 exposure-induced hypertension. In this study, we tested the hypothesis that hypertension caused by chronic exposure to low levels of E2 is mediated by central ET-1. To test this hypothesis, we used a previously established female rat model^22–24^.

## Materials and Methods

### Experimental animals and treatment

Adult female Sprague-Dawley rats (3–4 months old) purchased from Harlan, Indianapolis, IN were used in the experiments. They were housed in light (lights on between 5 am–7 pm) and temperature (23 ± 2 °C) controlled animal rooms and were provided food and water *ad libitum*. Experiments were performed in accordance with the NIH Guide for the Care and Use of laboratory animals in research and were approved by the Institutional animal care and use committee at Michigan State University. In experiment 1, we assessed the role of central ET_A_ receptor in mediating chronic E2-induced increase in arterial pressure. Animals were implanted with subcutaneous radiotelemeters for continuous recording of blood pressure as described previously^7^. Control blood pressure measurements were recorded for 5 days. The animals were then divided into two groups (n = 8/group) and were either sham-implanted (controls) or implanted with 90 day slow-release E2 pellets (20 ng/day, Innovative Research America, Sarasota, FL) subcutaneously. After 75 days of E2 exposure, the animals were subdivided further into 4 groups (n = 4/group) and implanted with an i.c.v. cannula in the lateral ventricle by stereotaxic surgery. Briefly, animals were anesthetized with pentobarbital and placed in a stereotaxic frame. The co-ordinates for the lateral ventricle were 0.3 mm posterior, 4 mm lateral and 3.4 mm ventral (depth) to the bregma. The skull was exposed and a hole was drilled and a cannula attached to an Alzet minipump (Model 2002; Alzet Osmotic Pumps, Cupertino, CA) was inserted through the hole and held in place by dental cement. The pump was slid through the incision into a subcutaneous pocket on the animal’s back. The pump was charged with either artificial cerebrospinal fluid (aCSF) or aCSF containing BQ-123 so that it released 400 pMol of the drug/hour at a flow rate of 0.5 μl/hour. Animals in groups 1 and 2 were sham implanted and E2 implanted respectively and received an Alzet pump charged with aCSF. Animals in groups 3 and 4 were sham implanted and E2 implanted respectively and received an Alzet pump charged with BQ-123. The minipumps were in operation for 2 weeks. By the end of these 2 weeks, E2 implanted animals were at the end of 90 days of E2 exposure and were euthanized with corresponding controls. Body weight was obtained at the time of sacrifice. The heart and kidneys were removed and weighed.

In experiment 2, 3–4 months old female SD rats were divided into 2 groups (n = 4–5/group), sham-implanted (controls) or implanted subcutaneously with 90-day slow-release E2 pellets (20 ng/day, Innovative Research America, Sarasota, FL). After 90 days of treatment, animals were euthanized and brains and brain stem were collected and stored at −80 °C until further analysis. Trunk blood was used to measure estradiol levels using radioimmunoassay as described previously^25^. Brains were sectioned (300 µm thickness) using a cryostat (Slee-Mainz, London, UK). The sections were placed on a cold stage maintained at −10 °C and the PVN and RVLM were microdissected as described previously using Palkovits’ microdissection technique^23^. Tissue punches were used for western blotting, RNA extraction and quantitative RT-PCR as described below.

### Quantitative RT-PCR

#### RNA extraction and cDNA synthesis

RNA was extracted from the RVLM and PVN punches using MELT Total Nucleic Acid Isolation System (Ambion Inc, Austin, TX) according to the manufacturer’s instructions. The tissue was digested using the Multi-Enzymatic Liquefaction of Tissue (MELT) mix provided in the kit. The RNA was eluted in a volume of 500 µl, after on-bead Turbo DNAse digestion (Ambion Inc, Austin, TX). The quality of the RNA was assessed using a Nanodrop spectrophotometer prior to cDNA synthesis. First strand cDNA was synthesized by reverse transcribing 400 ng of total RNA using RT^2^ First Strand Kit (SABiosciences, Frederick, MD).

#### qRT-PCR analysis

The cDNA synthesized from RVLM and PVN samples were used to perform quantitative real-time PCR. RT^2^ Real-Time PCR SYBR Green/ROX Master Mix (SABiosciences, Frederick, MD), cDNA samples, and the appropriate amount of RNAase-free water were combined. Each reaction contained 12.5 μL of PCR master mix, 2 μL of cDNA, 1 μL each of forward and reverse primer and 8.5 μL of water. The total reaction volume was 25 μL. The forward and reverse primers for ET-1, ET_A_, and ET_B_ were purchased from Integrated DNA Technologies (Coralville, IA) and are provided in Table 1. The reactions were performed in an Applied Biosystems 7500 Real-Time PCR System (Applied Biosystems) with the following run method: 50 °C for 2 min, 95 °C for 2 min, followed by 40 cycles of 95 °C for 15 sec, 60 °C for 60 sec and 72 °C for 35 sec. At the end of amplification, a melting curve analysis was done by heating the PCR products to 65–95 °C and held for 15 sec at increments of 0.2 °C, and the fluorescence was detected to confirm the presence of a single amplification product. After obtaining the CT values, the values were compared between the control and treatment group according to 2^−ΔΔCT^ method.

**Table 1:** Primer sequences for Real Time RT-PCR.

Gene	Forward Primer	Reverse Primer	Product Size (bp)
ET-1	TCTTCTCTCTGCTGTTTGTGGCTT	TCTTTTACGCCTTTCTGCATGGTA	407
ET_A_	AGTGCTAATCTAAGCAGCCAC	CAGGAAGCCACTGCTCTGTAC	491
ET_B_	AGCTGGTGCCCTTCATACAGAAGGC	TGCACACCTTTCCGCAAGCACG	919
β-actin	CGTAAAGACCTCTATGCCAA	AGCCATGCCAAATGTCTCAT	351

### Western blotting for ET_A_ receptor

The PVN and RVLM punches were solubilized in lysis buffer [0.5 mmol/l Tris·HCl (pH 6.8), 10% SDS, and 10% glycerol] with protease inhibitors (0.5 mmol/l PMSF, 10 g/l aprotinin, and 10 g/l leupeptin). An ultrasonic processor was used to homogenize punches (1–2 s pulses, with intermediate vortexing), which were centrifuged for 10 min at 5,000 rpm at 4 °C. The supernatant was collected, and protein concentration was determined using a bicinchoninic acid protein assay (Pierce, Rockford, IL). Proteins (4:1 dilution in denaturing sample buffer, boiled for 5 min) were separated on precast SDS-polyacrylamide gels (Pierce, Rockford, IL) and transferred to Immobilon-P membranes. Membranes were blocked for 3 h in blocking buffer containing Tris-buffered saline-Tween (TBS-T), 4% chick egg ovalbumin, and 2.5% sodium azide. Blots were probed overnight at 4 °C with polyclonal rabbit anti ET_A_ antibody (1:200 dilution; Alomone Labs, Israel) and monoclonal mouse β-tubulin antibody (1:1,000 dilution; Millipore; Temecula, CA), washed and incubated with the appropriate secondary antibodies for 1 h at 4 °C. The ET_A_ antibody had been tested previously with appropriate positive controls in the laboratory. Blots were then incubated with enhanced chemiluminescence (ECL) (Fisher Scientific, Pittsburgh, PA) reagents for visualization of the bands. The intensity of the bands was measured using NIH’s Image J software.

### Statistical analysis

All statistical procedures were performed using STATVIEW software (JMP Statistical Discovery, Cary, NC). Changes in MAP, HR, SBP and DBP profiles before BQ123 or ACSF administration were analyzed by repeated measures ANOVA followed by Bonferoni-Dunn test. The average values were compared using one-way ANOVA followed by student’s t-test. Differences in profiles of blood pressure and heart rate after day 75 were analyzed using repeated measures ANOVA and differences in average BP parameters were analyzed by ANOVA. The differences in serum estradiol, fold change in gene expression and protein levels from western blotting were analyzed by unpaired student’s t-test. A *p*-value of <0.05 was considered statistically significant.

## Results

### Estradiol pellet implantation increases serum estradiol levels

**Figure 1:**
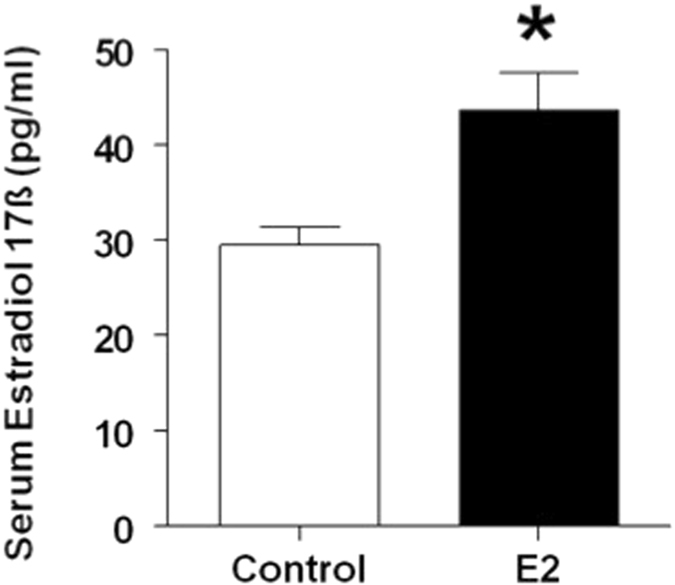
Serum estradiol levels in control and E2 treated rats. Adult female Sprague Dawley rats were either sham-implanted or implanted with slow release estradiol 17-β (E2) pellets for 90 days. Estradiol levels were measured by RIA in serum collected from trunk blood at the time of sacrifice at the end of 90 day exposure. *Indicates p < 0.01.

Estradiol levels (pg/ml; Mean ± S.E.) in serum from trunk blood were 29.54 ± 1.9 in control animals and increased significantly to 43.73 ± 3.9 in E2 pellet implanted animals (p = 0.0092) (Fig. 1).

### Chronic E2 exposure increases arterial pressure

**Figure 2:**
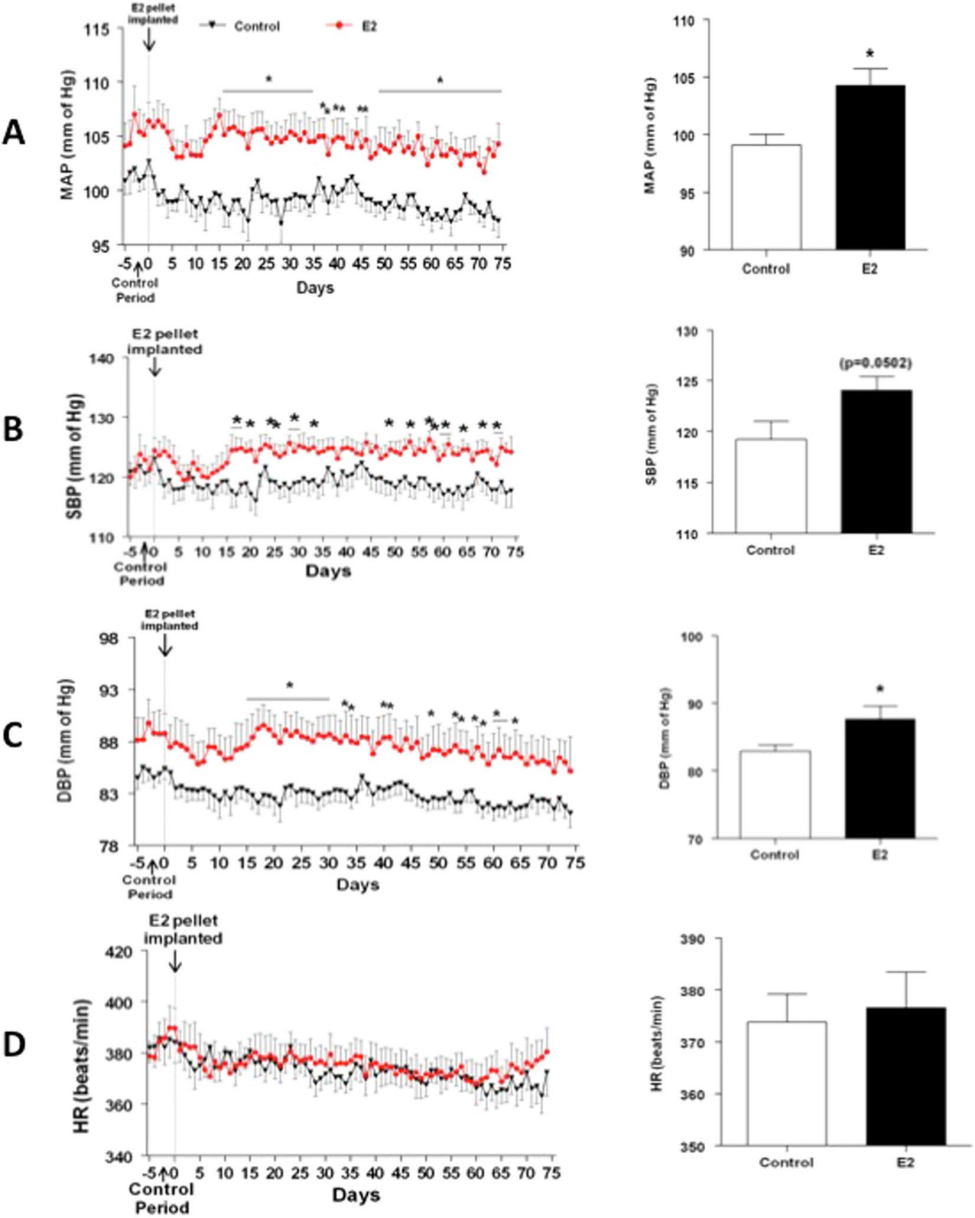
Time course for chronic E2 exposure-induced changes in blood pressure parameters. Line graphs depicting mean arterial pressure (MAP; mmHg) (**A**), systolic blood pressure (SBP) (**B**), diastolic blood pressure (DBP; mmHg) (**C**) and heart rate (HR; beats/min) (**D**): closed red circles represent E2 pellet implanted (20 ng/day, 90-day slow-release pellets) and closed black triangles represent control rats (n = 7–8/group). Panels on the right: Bar graphs showing the average values of the cardiovascular parameters over the entire observation period. *Denotes significant difference (p < 0.05) from control rats.

In order to determine the time course of E2-induced increase in arterial pressure, blood pressure recordings were obtained from day 0 of E2 exposure. The daily average profiles and the overall average mean arterial pressure (MAP), systolic blood pressure (SBP), diastolic blood pressure (DBP) and heart rate (HR) starting from day 1–75 in sham and E2-treated rats are shown in Fig. 2(A–D). The MAP (Mean ± SEM, mmHg) in sham animals was about 100.9 ± 1.2 prior to implantation and remained unchanged over the entire period of observation. MAP in the E2 group was not different from the control group during the pretreatment period but remained elevated after about 15 days (p < 0.05; Fig. 2A left panel). The average MAP measured during the 75 days of observation in control rats was 99.04 ± 0.8. On the other hand, E2 exposure increased average MAP significantly to 104.37 ± 1.4 (p = 0.0057; 2A Right panel). Similarly, the SBP and DBP profiles in E2-treated were significantly elevated in E_2_-treated rats compared to control rats (p < 0.05; Fig. 2B and C left panels). E2 exposure also significantly increased the average SBP and DBP (Mean ± SEM, mmHg; 123.4 ± 2.2 and 87.6 ± 1.92 respectively) compared to control rats (121.1 ± 2.2 and 82.8 ± 0.89 respectively; p = 0.0502 and p = 0.0341 respectively; Fig. 2B and C right panels). There were no marked differences in HR profiles between control and E2 treated animals (Fig. 2D).

### Chronic E2 exposure increases ET-1 and ET_A_ receptor gene expression in the PVN and RVLM

**Figure 3:**
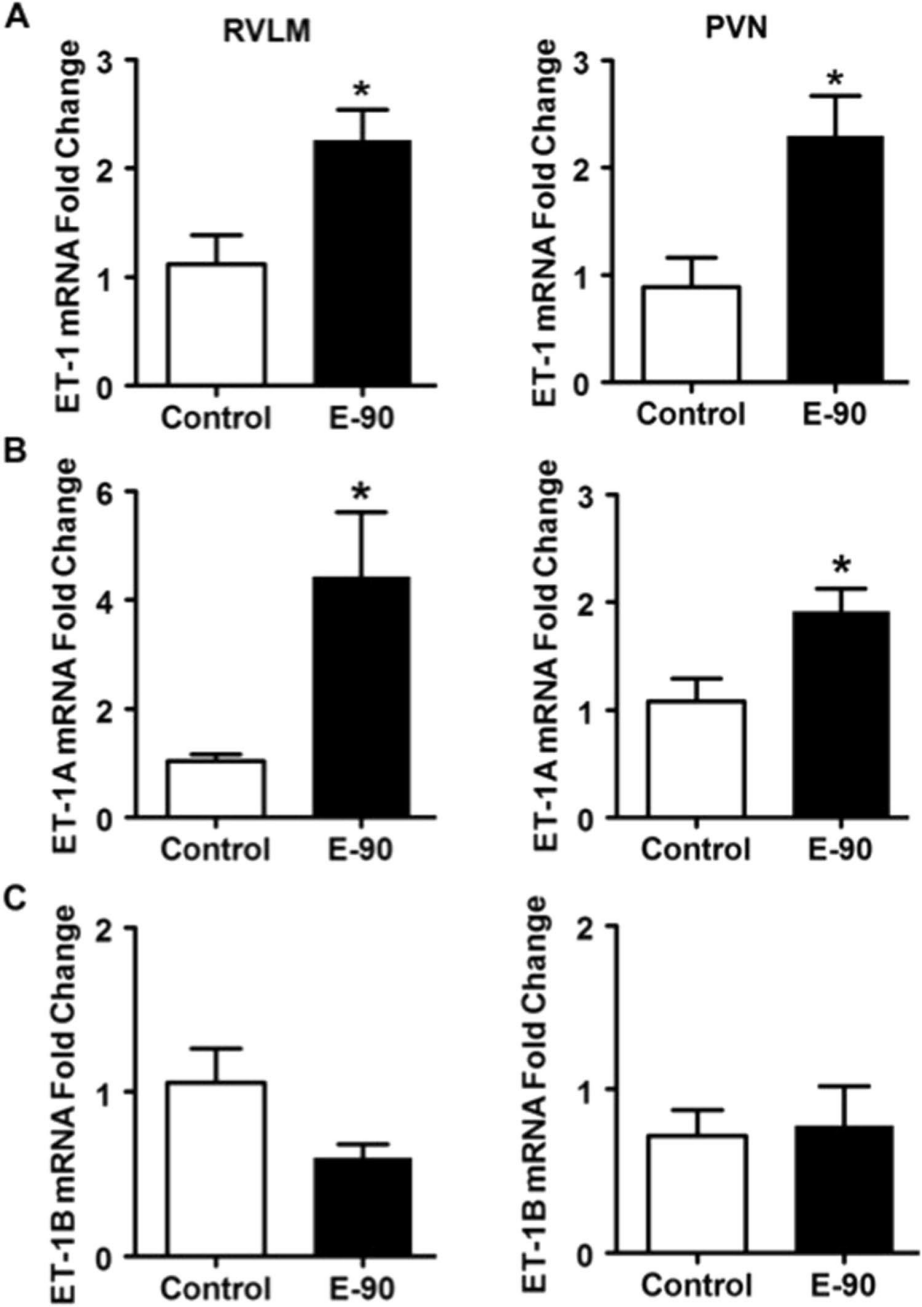
Effect of chronic E2 exposure on the gene expression of ET-1 and its receptors in the RVLM and PVN. The mRNA expression levels of ET-1, ET_A_ and ET_B_ receptor and Angiotensin II type 1 receptor in the RVLM and PVN of control and E2 treated rats are shown in (**A**–**H**). The fold change was calculated relative to β-actin by the comparative C_t_ method using 2^−ΔΔCt^. The C_t_ values of all the groups were normalized to control rats (n = 4–5 per group). *Denotes significant difference (p < 0.05) from control group.

Chronic E2 exposure resulted in 2-fold up-regulation in the gene expression (Fold change relative to control; Mean ± SEM) of ET-1 in the RVLM (2.25 ± 0.29) and PVN (2.29 ± 0.37) compared to controls (p = 0.03) (Fig. 3A). Similarly, the gene expression of ET1_A_ receptor was also significantly up-regulated in the RVLM (4.43 ± 1.2) and PVN (1.92 ± 0.21) of E2 treated animals compared to the controls (p = 0.02) (Fig. 3B).

**Figure 4:**
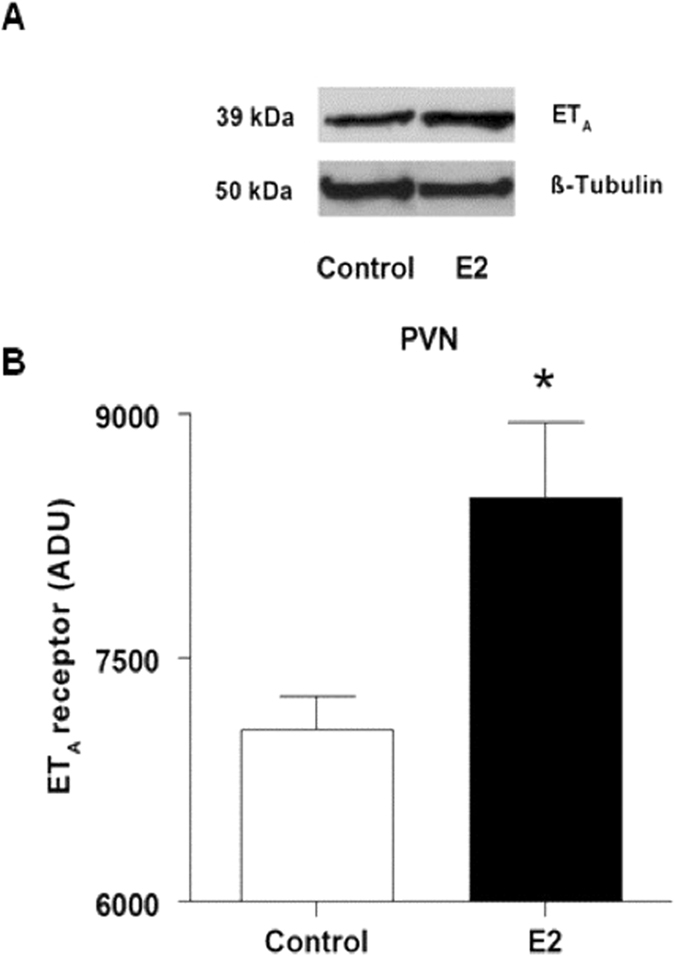
Effect of chronic E2 exposure on protein levels of ET_A_ in the PVN. Sample blots and densitometry results from western blot analysis of ET_A_ in the PVN of control and E2-treated rats are shown. Bar graphs represent mean ± SE for 4–5 animals. *Indicates significant difference from control animals.

In correlation with changes in mRNA levels, the protein levels of ET1_A_ receptor were also significantly higher in the PVN of E2 treated animals (Fig. 4A and B). However, ET1_A_ protein levels in the RVLM were below detectable limits.

### ET_A_ receptor antagonist (BQ-123) reverses chronic E2-induced hypertension

**Figure 5:**
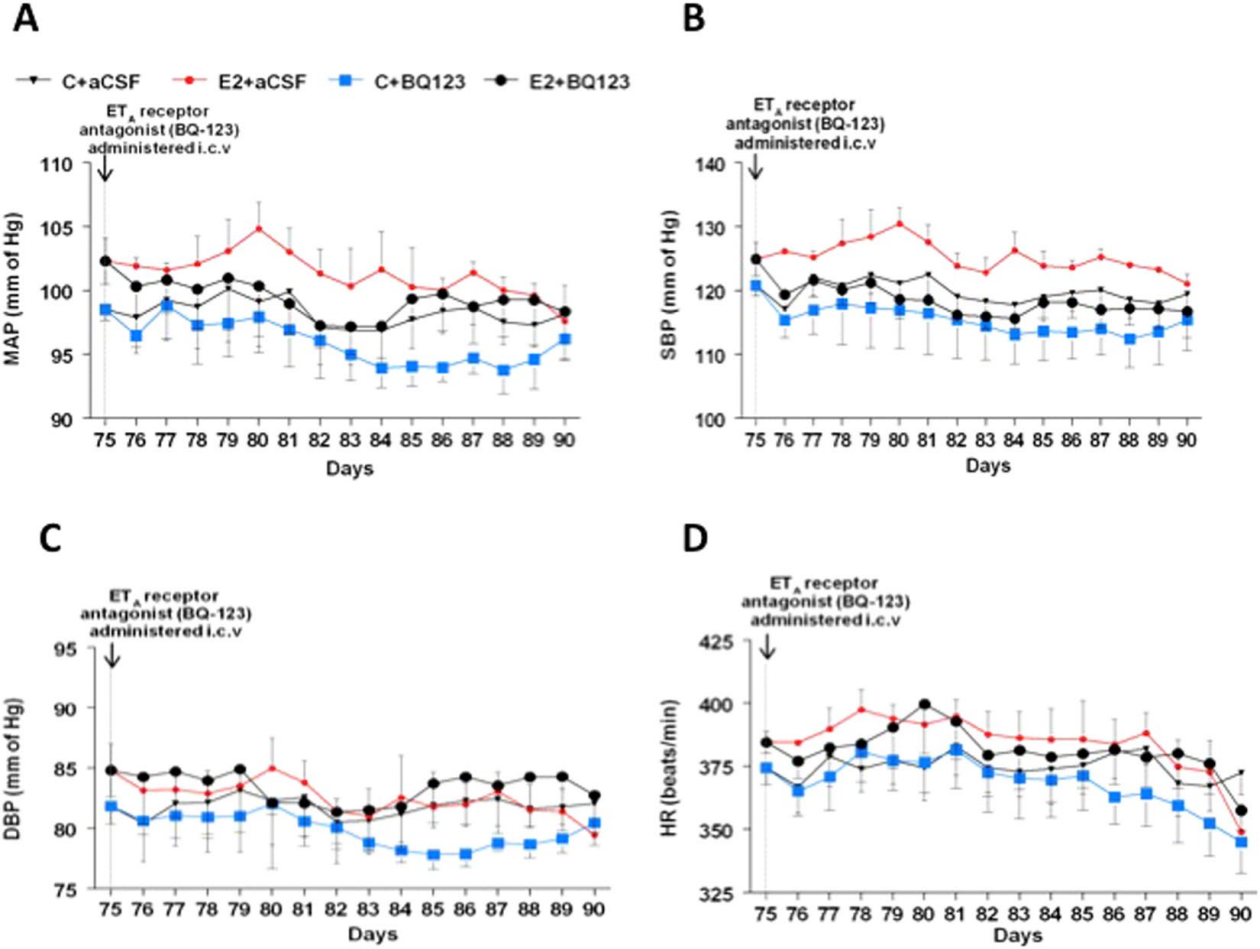
Effect of ICV ET_A_ antagonist on chronic E2-induced increase in MAP. (**A**–**D**): Line graphs depicting MAP (mmHg), systolic blood pressure (SBP), diastolic blood pressure (DBP; mmHg) and heart rate (HR; beats/min) respectively: closed circles in red represent E2 pellet implanted (20 ng/day, 90-day slow-release pellets) treated with aCSF (E2 + aCSF) and closed triangles represent control SD rats treated with aCSF (C + aCSF), Blue squares represent control rats treated with ET_A_ antagonist (BQ-123) (C + BQ-123) and closed circles in black represent E_2_ pellet implanted (20 ng/day, 90-day slow-release pellets) rats treated with BQ-123 (E2 + BQ-123). Inverted arrow indicates the day ET_A_ receptor antagonist (BQ-123) administered i.c.v.

The daily average profiles and the average mean arterial pressure (MAP), systolic blood pressure (SBP), diastolic blood pressure (DBP) and heart rate (HR) from day 75–90 of E2 treatment for Sham + aCSF, E2 + aCSF, Sham + BQ-123 and E2 + BQ-123 rats are shown in Fig. 5(A–D). ICV administration of BQ-123 appeared to produce modest reductions in MAP and SBP, but these were not significantly different from the E2 + aCSF group. There were no changes in average BP parameters in the 4 groups during the entire period of observation (data not shown). There were no significant changes in body weight, heart weight or kidney weight among the different treatment groups (Table 2).

**Table 2:** Body weight, heart and kidney weight in animals that were sham-implanted or implanted with E2 pellets and infused with ACSF or BQ123 i.c.v.

Parameter	Control + ACSF	E2 + ACSF	Control + BQ123	E2 + BQ123
Body weight (g)	269.3 ± 17.3	248.9 ± 3.7	254.6 ± 9.6	267.02 ± 8.5
Heart weight (g)	0.906 ± 0.05	0.952 ± 0.08	0.907 ± 0.06	0.895 ± 0.04
Kidney weight (g)	1.705 ± 0.12	1.671 ± 0.05	1.637 ± 0.18	1.834 ± 0.11

## Discussion

Previously, we had demonstrated that chronic exposure to low levels of E2 increases blood pressure in female Sprague Dawley rats and that this effect was most probably mediated through increases in superoxide levels in the RVLM^7^. In concordance with that study, we have found that chronic E_2_ exposure increased MAP, HR, SBP and DBP in intact female SD rats. In addition, in the present study, we were able to monitor blood pressure from the beginning of E2 exposure and observed that E2 treatment increased MAP starting as early as 2 weeks after E2 treatment. E2 exposure also increased the transcript levels of ET-1 and ET_A_ receptor, but not the ET_B_ receptor, in both the RVLM and PVN. We also observed an increase in ET_A_ protein levels in the PVN of E2 treated animals, but not in the RVLM. Intracerebroventricular (i.c.v.) administration of an ET_A_ receptor antagonist, BQ-123 did not completely block the E2-induced increase in MAP. Taken together, these results suggest that the possibility that increased brain ET-1 activity may contribute to increases in arterial pressure associated with chronic E2 exposure.

Several studies support our findings on the role of central ET-1 in the development of hypertension. Increase in ET-1 levels in the brain has been reported paralleling increases in MAP in DOCA-salt hypertensive rats^19^. Also, Rossi *et al*. reported that i.c.v. administration of ET-1 increased MAP in a dose-dependent manner in Long-Evans rats^25^ and Sprague Dawley rats^26^, while others have also demonstrated the same in SHR and SHR-SP rats^18^. Lesioning of the PVN prevents central ET-1-induced increase in blood pressure^10^. Further, microinjection of ET-1 bilaterally in the PVN stimulated the cardiac sympathetic afferent reflex, increased MAP and renal sympathetic nerve activity^21^. Taken together, these studies indicate that ET-1 levels in the PVN play an important role in blood pressure regulation. In contrast to the effects on the PVN, the pressor effect of ET-1 injections into the RVLM have been variable^27^. In one study, i.c.v. ET-1 was found to activate vasomotor neurons in the RVLM^28^. While in another study, injection of ET-1 in the RVLM produced an initial increase in blood pressure followed by a prolonged hypotensive response^27^.

Other studies have shown that i.c.v. ET_A_ but not ET_B_ receptor blockade reversed ET-1 induced increases in blood pressure^18, 25^. A similar effect was observed when BQ-123 was microinjected into the PVN as well^21^. In the present study, however, i.c.v administration of BQ-123 failed to completely block E2’s effects on BP parameters. The time of BQ-123 administration could have played a role in this effect. In the present study, BQ-123 was administered towards the end of E2 exposure and it is likely that the effect of E2 was beginning to fade as can be seen by the gradual lowering of BP profiles in the E2 + aCSF group (Fig. 5). However, this was the first study in which we attempted to monitor BP changes from the beginning to the end of E2 exposure and earlier administration of BQ-123 could have provided better insight into this phenomenon.

ET-1 could induce hypertension in our model through a few mechanisms. In the periphery, ET-1 has been reported to increase superoxide production via an NADPH oxidase dependent mechanism in the vasculature of DOCA-salt hypertensive rats^29^. A similar mechanism could be in operation in the brain as well. Since superoxide production increases in the RVLM of E2-treated rats^7^ and ET-1 expression is higher in both the RVLM and PVN of E2-treated animals there is a likelihood that ET-1 might activate NADPH oxidase to induce superoxide production in our model. Although we did not measure superoxide production or NADPH oxidase expression in these studies, we have shown that superoxide levels^7^ and NADPH oxidase gene expression do increase in the RVLM of rats chronically exposed to low doses of E2^30^. This is supported by a study in which microinjection of ET-1 in the PVN increased superoxide production and this effect was blocked by BQ-123^21^. Moreover, superoxide scavengers such as tempol and PEG-superoxide dismutase were able to block the increase in blood pressure caused by PVN microinjections of ET-1^21^.

The source of ET-1 in the brain is not clear. Although brain vasculature could be an important source of ET-1, it is reported to be synthesized by glial cells as well^31^. Presence of estrogen receptors in glial cells and the fact that chronic E2 exposure has been previously shown to cause gliosis^22^ allows us to speculate that chronic E2 exposure activates glial cells to release ET-1, which could in turn, act on adjacent neurons and glial cells in a paracrine manner. Further studies are needed to investigate this possibility.

In conclusion, our studies provide evidence that chronic E2-induced increase in MAP in young Sprague-Dawley female rats is mediated through central ET-1, possibly by acting through ET_A_.
